# Acute puerperal uterine inversion with successful manual transvaginal repositioning: A case report

**DOI:** 10.1097/MD.0000000000037986

**Published:** 2024-04-26

**Authors:** Qianqian Gao, Hong Jiang, Mengmeng Jia, Jinqiu Xiong

**Affiliations:** a Departments of Obstetrics, Weifang People’s Hospital, First Affiliated Hospital of Weifang Medical College, Weifang, Shandong, China; b Departments of General Surgery, Weifang People’s Hospital, First Affiliated Hospital of Weifang Medical College, Weifang, Shandong, China.

**Keywords:** case report, complication in childbirth, puerperal, reposition, uterine inversion

## Abstract

**Rationale:**

Uterine inversion is a rare medical condition that is categorized as puerperal and nonpuerperal. Repositioning of uterine involution can be done manually or surgically, the latter of which involves abdominal manipulation and disruption of the integrity of the uterine wall, which can lead to complications for the patient in subsequent pregnancies, such as uterine rupture.

**Patient concerns:**

We report a case of acute puerperal uterine inversion that was manually repositioned transvaginally. An ultrasonogram and reset schematic were also presented. A 23-year-old woman (gravida 1 para 0) was admitted to the hospital with a full-term pregnancy.

**Diagnoses:**

In the postpartum period, we found placental adhesions and uterine inversion into the uterine cavity, which was confirmed by bedside ultrasound.

**Interventions and outcomes:**

We administered analgesic, relieving uterine spasms, and antishock therapy along with manual stripping of the placenta and ultrasound-guided uterine repositioning. After successful repositioning the patient vaginal bleeding decreased rapidly and she was discharged 3 days after delivery.

**Lessons:**

Early recognition, antishock therapy and prompt repositioning are key in the management of puerperal uterine inversion. We hope that this case will enable clinicians to better visualize the ultrasound imaging of uterine inversion and the process of manual repositioning.

## 1. Introduction

Uterine inversion is the protrusion of the uterine fundus into the uterine cavity, the cervical os, or out of the vagina, and can be categorized as puerperal or nonpuerperal. Uterine involution can be categorized as acute (<24 hours), subacute (between 24 hours and 1 month), and chronic (>1 month) depending on the timing of its occurrence in relation to delivery.^[1]^ There is a wide disparity in the incidence of puerperal uterine involution, ranging from 0.5 to 2.9 per 10,000^[2,3]^, while the incidence of nonpuerperal uterine involution is even lower.^[3]^ Puerperal uterine inversion occurs mostly after transvaginal delivery and has been reported in a few cases during cesarean section.^[4]^ Puerperal inversion of the uterus is one of the life-threatening obstetric emergencies as it causes postpartum hemorrhage requiring blood transfusion and may lead to hypovolemic shock.^[2]^ The possibility of puerperal uterine inversion should be considered in puerperal patients presenting with irregular vaginal bleeding, abdominal pain, hypovolemic shock, and vaginal masses. We present a case of successful ultrasound-guided manual reversal of puerperal uterine inversion. Obstetricians should be alert and ready to respond to this rare clinical condition. Maintaining hemodynamic stability and timely reduction is the key to successful rescue. We hope that this article will provide clinicians with a better understanding of the clinical manifestations of uterine inversion and possible treatment options from this case report.

## 2. Case presentation

**Figure 1.**
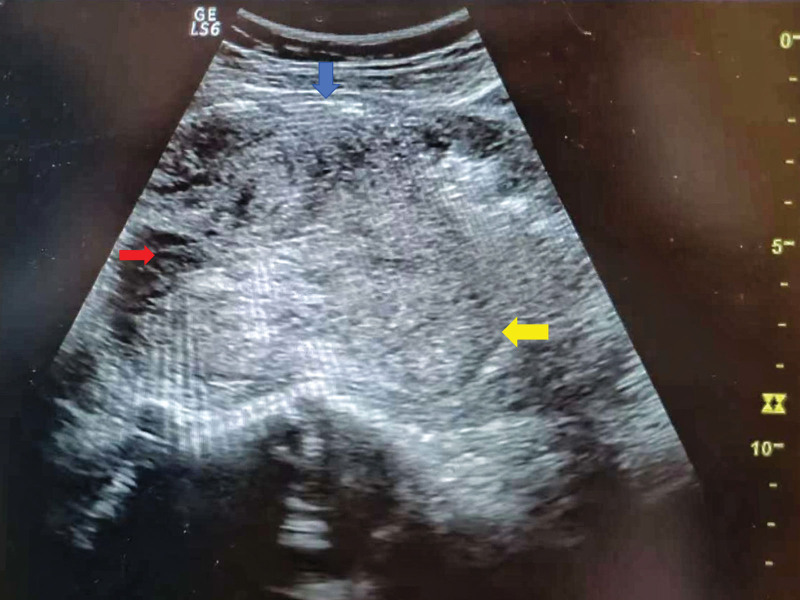
Uterine inversion-ultrasound imaging. Blue arrow: plasma layer of the anterior uterine wall; Red arrow: cupping structure formed after inversion of the uterus; Yellow arrows: fundus tissue that inverts into the uterine cavity.

**Figure 2.**
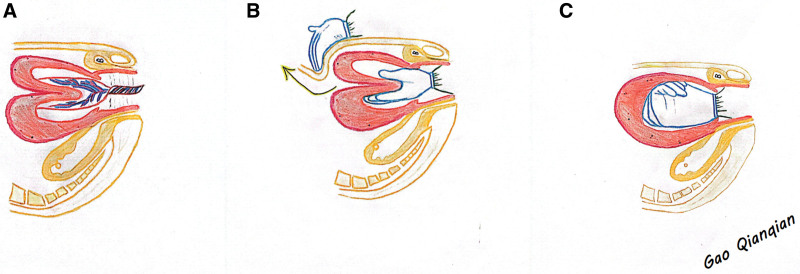
Diagram of uterine inversion and repositioning: (A) Uterine inversion and placenta adhesion. (B) The fundus and body of the uterus, which has turned inward to the cervical os, are held in the palm of the right hand. Force is applied uniformly and slowly in a parallel direction along the pelvic axis, and the left hand is pressed against the fundus of the patient uterus through the abdomen, so that the portion of the inwardly-turned uterus near the cervix of the uterus is the first to be returned to the uterus. (C) After resetting the uterus to its normal anatomical structure, the author right hand was placed in the shape of a fist against the fundus of the uterus and administered hysterotonin to promote contractions. As the uterus coordinated contractions, the author slowly withdrew his right hand.

A 23-year-old woman (gravida 1 para 0) was admitted to the hospital with a full-term pregnancy. The patient was conceived naturally, received regular prenatal care during her pregnancy, and went through the entire period without incident. The patient denied uterine fibroids and other gynecological disorders. She had no significant medical history, denied smoking, denied a family history, and with body mass index, 22.1 kg/m^2^. She started contractions spontaneously and underwent intraspinal anesthesia for pain relief. Due to the patient fatigue and weak contractions in the second stage of labor, 2.5 units of oxytocin were applied intravenously to enhance contractions. The patient delivered a live-born infant with an Apgar score of 10/10/10 (1, 5, 10 minutes) and weighing 2910 grams. After the delivery of the fetus, to prevent postpartum hemorrhage, 10 units of oxytocin were given intravenously. Physical examination revealed the placenta was at the cervical os after 17 minutes. No normal uterine structures were palpated on abdominal examination. It was found that the inverted fundus of the uterus protruded into the uterine cavity. Bedside ultrasound confirmed uterine inversion (Fig. 1). Combined with the patient past history of disease, physical examination, and ultrasound findings, we excluded the diagnosis of uterine fibroids. The patient blood pressure was 102/59 mm Hg and pulse was 103 beats per min. The placenta remained adherent. We gave an injection of pethidine hydrochloride 100 mg. After manually stripping the placenta, we performed the first repositioning under ultrasound guidance, but the repositioning failed because of the uterine contraction ring encountered in the lower segment of the uterus. Along with the first repositioning we gave an injection of magnesium sulfate 5 g, rapid infusion of crystalloid and blood transfusion. We performed the second uterine repositioning under ultrasound guidance. The patient blood pressure was 88/51 mm Hg and pulse was 134 beats per min. Successful reduction was achieved 35 minutes later (Fig. 2). The patient blood pressure was 79/52 mm Hg and pulse was 140 beats per min. Total blood loss was estimated to be 1200 mL. The patient vaginal bleeding decreased rapidly after uterine repositioning. After successful reduction, 10 units of oxytocin were administered intravenously. We infused a total of 400 mL of red blood cell suspension. The patient cooperated well during the repositioning procedure as we gave adequate analgesic and relieved uterine spasm. The patient was treated with antibiotics and pro-contractile agents after delivery, had good uterine contractions and was discharged from the hospital after 3 days. The patient was discharged with a hemoglobin of 96 g/L (118 g/L before delivery). At her follow-up visit at 6 weeks, ultrasonography suggested that the patient uterus was recovering well. And the patient denies abnormal vaginal bleeding.

## 3. Discussion

Uterine inversion is an extremely rare condition that occurs primarily during the peripartum period and sometimes during the nonpartum period, with the youngest patient reported being 11 years of age.^[5]^ Placental abnormalities are the strongest risk factor for uterine inversion, followed by prolonged labor.^[2]^ Postpartum uterine wall relaxation, cervical dilatation, placental adherence, short umbilical cord, sudden uterine emptying, midwife compression of the uterine fundus, or excessive pulling of the umbilical cord when the placenta is not stripped, can induce uterine inversion.^[6]^ In the case of nonpuerperal uterine inversion can be due to outward growth of uterine submucosal fibroids,^[7]^ endometrial polyps,^[8]^ and uterine malignant tumors^[9–12]^ (e.g., endometrial carcinoma, rhabdomyosarcoma), which result in the uterine fundus turning out. Depending on the degree of inversion, it can be categorized into 4 degrees. One degree of inversion occurs when the body or wall of the uterus extends to the cervix but does not protrude beyond the cervical ring. In second-degree inversion, the body of the uterus passes through the cervical ring but does not reach the perineum. In third-degree inversion, the fundus of the uterus extends to the perineum, and, finally, if the vagina, together with the uterus, inverts beyond the perineum, it is regarded as fourth-degree inversion.^[2]^

Patients with uterine inversion may present with unexplained irregular vaginal bleeding, abdominal pain,^[8]^ traumatic shock or hemorrhagic shock.^[13]^ Physical examination demonstrated: Vaginal palpation: Palpable inversion of the uterine fundus. Abdominal palpation: Normal fundal uterine tissue cannot be palpated, or the cupping structure may be palpable. If the diagnosis cannot be confirmed by physical examination, bedside ultrasound needs to be utilized as soon as possible.^[14,15]^ Typical ultrasound images show a transabdominal ultrasound cross-section of the uterus showing a “target sign” (or “bull-eye sign”), that is, the uterine fundus is highly echogenic centered on the peripheral uterine wall, which is hyperechoic, and in the sagittal plane suggests that the uterine fundus is collapsing into the uterine cavity.^[16]^ Some inverted uterus may be misdiagnosed as a cervical tumor or submucosal fibroid prolapsing out of the uterine cavity, and in cases of chronic uterine inversion, magnetic resonance imaging is the key to diagnosis.^[5,17]^ T1-weighted imaging is not useful, T2-weighted imaging shows clear anatomy, and it is important to identify the round ligaments and fallopian tubes, which are pulled medially, along with the uterine fundus, into the U-shaped uterine cavity.^[18]^

About 83.4% of cases of postpartum uterine inversion are acute uterine inversion,^[19]^ which is one of the most important causes of postpartum traumatic shock, hemorrhagic shock and puerperal infections. Due to the urgency of the situation, early recognition, aggressive antishock therapy, relief of uterine cramps, and prompt repositioning were the keys to treatment.^[6]^ Depending on the degree of inversion and the presence or absence of infection, and the patient subsequent fertility requirements, different ways of repositioning are used.

This case occurred after a transvaginal delivery, and manual repositioning was the first step because of early recognition, the patient cervix was not yet closed, and her vital signs were stable. The patient was analgesic with intrathecal anesthesia during labor and delivery, and when uterine involution was found, we gave pethidine hydrochloride analgesic, magnesium sulfate to inhibit uncoordinated uterine contractions and relieve cervical spasms, and high-flow venous access for transfusion of blood and crystalloid fluids for antishock along with manual reset, recover to its normal anatomy. Ritodrine or terbutaline may also be used to relieve uterine spasms.^[20]^ The lesson learned from our cases is that if placental adhesions and uterine inversion are present at the same time, removing the adherent placenta first to avoid pushing the larger tissue through the retracting ring will help to reposition the uterus. However, some literature suggests keeping the placenta in place until the uterus is repositioned to minimize bleeding.^[3,20]^ There is a case report of successful use of a Bakri-filled balloon for puerperal uterine involution during cesarean section.^[21]^ O’Sullivan hydrostatic reduction has also been reported for acute uterine inversion.^[22]^ We recommend that uterine repositioning be done under ultrasound guidance, which helps to visualize and guide the uterine repositioning. In addition, reverse traction on the cervix or abdominal pressure against the inverted cervical ring can be used to help with successful repositioning. A 14-year retrospective study suggests that transabdominal ultrasound confirmation of placental abruption may help prevent postpartum uterine inversion.^[23]^

If these measures are ineffective, immediate surgical repositioning should be performed, including open manual repositioning,^[24,25]^ laparotomy, and laparoscopic surgery. Operative procedures used include Huntington procedure, Haultain procedure, Kustner approach, etc, and some patients may need to undergo hysterectomy.^[7,9,26,27]^ Laparoscopic visualization is feasible and safe in cases of nonpuerperal uterine involution with undetermined pathology.^[9]^ Robot-assisted laparoscopic supracervical repair for chronic puerperal uterine inversion preserves the integrity of the cervix and may reduce adverse effects on future pregnancies.^[28]^ If the patient undergoes surgical correction of uterine inversion, it is safer to opt for cesarean section in subsequent pregnancies, taking into account that the uterine fundus or corpus has been incised.^[6]^

It is recommended that after successful repositioning uterine contractions be facilitated by the administration of uterotonics and that the operator hand be kept in the uterine cavity and withdrawn when the uterus has contracted in order to avoid recurrence of uterine inversion, and that a balloon be used as well.^[3]^ Considering that uterine inversion increases the risk of infection, it is recommended that antibiotics be given after delivery to prevent infection.^[1]^ It is also important to be alert for lethal air embolism during endometrial repositioning of the uterus.^[29]^ Our article describes in detail the diagnostic and therapeutic process of acute puerperal uterine inversion, and provides detailed ultrasound images and schematic diagrams for easy visualization by clinicians. However, because of the urgency of the situation, we did not retain the video data of the examination images and manual repositioning of uterine inversion.

Uterine inversion is a rare delivery complication that can cause severe postpartum hemorrhage and shock. Early recognition and early repositioning can prevent serious consequences, and obstetricians should be alert and ready to respond to this rare clinical condition. Maintaining hemodynamic stability and timely reduction is the key to successful rescue.

## Author contributions

**Data curation:** Hong Jiang, Mengmeng Jia.

**Resources:** Hong Jiang, Mengmeng Jia.

**Supervision:** Jinqiu Xiong.

**Visualization:** Qianqian Gao.

**Writing – original draft:** Qianqian Gao.

**Writing – review & editing:** Jinqiu Xiong.
